# Under-Recognition of Fractures as Osteoporosis Indicators

**DOI:** 10.3390/geriatrics4010009

**Published:** 2019-01-09

**Authors:** Violet S. Lagari, Fatima Al-Yatama, Gracielena Rodriguez, Hara R. Berger, Silvina Levis

**Affiliations:** 1 Division of Endocrinology, Diabetes, and Metabolism, University of Miami Miller School of Medicine, 1400 NW 10th Ave Suite 807 Miami, FL 33136, USA; gracielena.rodriguez@jhsmiami.org; 2 Endocrinology Section, Miami Veterans Affairs Healthcare System, 1201 NW 16th St (11GRC), Miami, FL 33125, USA; 3 Department of Medicine, Farwaniya Hospital, P.O. Box 13373, Farwaniya 81004, Kuwait; falyatama@gmail.com; 4 Reproductive Health Physicians, 4675 Ponce de Leon Blvd. Suite 204, Miami, FL 33146, USA; drberger@repromd.com; 5 Department of Medicine, University of Miami School of Medicine, 1600 NW 10th Ave. Suite 1140 Miami, FL 33136, USA; slevis@med.miami.edu; 6 Geriatric Research Education and Clinical Center, Miami Veterans Affairs Healthcare System, 1201 NW 16th St (11GRC), Miami, FL 33125, USA

**Keywords:** osteoporosis, fracture, low-trauma fracture, veterans

## Abstract

After the first fracture, the risk of subsequent fractures increases significantly. Medical treatment can reduce the risk of a second fracture by about 50%, but many older adults do not receive osteoporosis medication following their first fracture. This observational study aimed to understand primary care management patterns of older adults after osteoporotic fractures at the Miami Veterans Affairs (VA) Healthcare System. A retrospective review of 219 fracture cases selected by International Classification of Disease (ICD-9) codes between October 2015 and September 2016 identified 114 individuals age ≥50 years who had a non-traumatic fracture code entered in their medical record for the first time. Among them, 72 (63%) did not undergo a bone mineral density (BMD) test or receive treatment in the 12 months following their fracture. Of the 40 individuals who had a BMD test post-fracture, 17 (100%) received or were considered for anti-osteoporosis treatment if their T-score indicated osteoporosis (T-score ≤−2.5), but only 8/23 (35%) if the T-score was >−2.5. Physicians are more likely to prescribe osteoporosis therapy based on a BMD T-score diagnosis of osteoporosis, rather than a clinical diagnosis of osteoporosis based on a low-trauma fracture. A change in practice patterns is necessary to decrease the incidence of fractures.

## 1. Introduction

Medical treatment rates of patients with osteoporotic fractures remain low despite the availability of several pharmacologic agents that reduce the risk of an initial fracture [1,2,3,4] and if started after the first fracture, also reduce the risk of subsequent fractures. Only 27% of women and 4% of men admitted with acute osteoporotic fractures are discharged with a prescription for an anti-osteoporosis medication, inclusive of calcium and vitamin D [5].

Osteoporosis is a public health problem affecting more than 200 million people worldwide, with combined costs in the United States (U.S.) estimated at $17 billion and growing [6,7]. Osteoporotic fractures are prevalent among the elderly and all major osteoporotic fractures are associated with increased mortality risk [8,9]. Although general awareness about osteoporosis and its consequences has increased steadily and effective treatments have been available for the more than two decades, drugs to reduce fracture risk are underutilized. Furthermore, rates of osteoporosis medication use after a fracture have declined significantly in the U.S., from 40.2% in 2002 to 20.5% in 2011 [10]. The lay press has contributed significantly to a disproportionate concern regarding the side effects of these medications [11]. 

Because having a history of fracture is a strong predictor of subsequent fractures, also known as secondary fractures [12], scientific organizations support the initiation of a Fracture Liaison Service to ensure adequate medical management and reduction of secondary fracture risk [13,14,15]. This approach has been successful when implemented in closed healthcare systems, as patients receive outpatient and inpatient care seamlessly. The Veterans Health Administration (VHA) is a closed healthcare system that cares for approximately nine million U.S. veterans, of which 3% are women and 64% are men aged 55 and older [16]. In 2012, the Office of the Inspector General at the Department of Veterans Affairs (VA) determined that only 42% of veterans with osteoporotic fractures are appropriately evaluated and treated by VA [17]. To evaluate the current need for a Fracture Liaison Service at VA, our goal is to evaluate post-fracture management patterns at one of the largest VA facilities. The objective of this study is to determine the proportion of patients without prior bone density testing or anti-osteoporosis treatment, who received adequate management after an osteoporotic fracture.

## 2. Methods

A VHA Central Data Warehouse report identified men and women 50 years of age or older who received primary care services at the Miami VA Healthcare System and had an International Classification of Disease (ICD-9) code for a low-trauma fracture entered in their medical record for the first time between 1 October 2015 and 30 September 2016. The codes included were 733.93, 733.96–733.98, 805, 807–813, and 820–824. The main outcome of interest was initiation of osteoporosis medication following a low-trauma fracture among those who had not been prescribed such medications prior to the fracture. A low-trauma fracture is defined as any fracture that excluded toes, metatarsal bones, fingers, metacarpal bones, skull, facial bones, and mandible, and was caused by a fall from standing height [12,18]. Each patient’s electronic medical record was reviewed by an endocrinologist who specializes in osteoporosis management. Cases were excluded if the chart review revealed that the fracture was pathologic, caused by high-impact trauma, or if the circumstance surrounding the fracture was unclear. Patients were also excluded if they received primary care outside of VA, if they resided in a non-VHA nursing home, if they had a life expectancy of less than 12 months, or were deceased at the time of the review.

The retrospective review of medical records included the identification of comorbidities, and use of tobacco and alcohol. Because our intent was to assess the extent of osteoporosis management in patients who had a fracture and were naïve of bone mineral density (BMD) testing and/or osteoporosis treatment, we also recorded any BMD testing and/or prescriptions of anti-osteoporosis medications in the 5 years before and 12 months after the fracture. This information identified and distinguished those patients who had never been assessed or treated for osteoporosis prior to the fracture from those who had. We compared these two groups to understand whether there were differences that may have led providers to screen one group earlier than another group for osteoporosis.

Baseline characteristics of the patients are described using proportions for categorical variables and means with standard deviations for continuous variables. Comparison of the demographic variables of the group who received management prior to and after the fracture was performed using Fisher’s exact test of proportions and independent samples *T*-tests. Statistical significance was assessed for *p* < 0.05. All analyses were performed using SPSS for Windows (Version 20.0; SPSS Inc., Chicago, IL, USA).

## 3. Results

**Figure 1:**
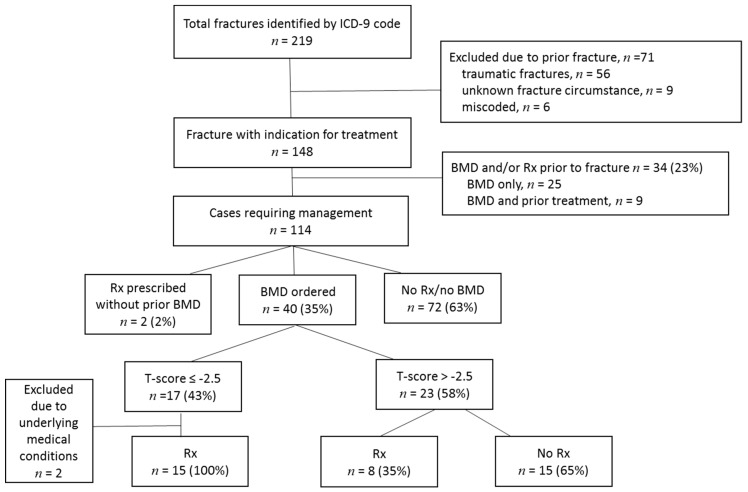
Study algorithm. BMD: bone mineral density; ICD: International Classification of Disease; Rx: prescription.

A total of 219 fracture cases were identified by ICD-9 codes during the study period. After exclusion of pathologic and traumatic fractures, cases where other conditions were miscoded as fractures, and cases where patients could not recall the circumstances surrounding their fracture, 148 patients were identified as having an indication to receive anti-osteoporosis therapy. As shown in Figure 1, 34/148 (23%) patients had received osteoporosis management prior to the fracture and were therefore excluded from the analysis. Management included having had a BMD test and/or treatment with osteoporosis medication up to five years before the fracture. All the patients with prior treatment were receiving a bisphosphonate. Thus, 114 patients were reviewed to understand post-fracture management practices in patients naïve of osteoporosis diagnostic testing and/or treatment.

Table 1 describes the demographic characteristics of the 114 patients included in this report. The demographics of the 34 patients who had osteoporosis management prior to the fracture (data not shown) were not statistically different regarding their gender, age, ethnicity, and type of fracture sustained, as compared with the group who did not receive management following the low-trauma fracture. Table 2 lists the site of fracture frequency; rib fracture was the most common type of low-trauma fracture sustained in this cohort, followed by radial fracture.

Among the 114 fracture cases, 72 (63%) patients did not have a BMD test or receive treatment in the 12 months following the fracture. Of the 40 individuals who had a BMD test after the fracture, 17 (100%) received or were considered to receive anti-osteoporosis medication consisting of bisphosphonates if their T-score indicated osteoporosis (T-score ≤−2.5), but only 8/23 (35%) were treated if the BMD result indicated a T-score >−2.5. A detailed chart review was performed to characterize the two patients who did not receive anti-osteoporosis therapy following the fracture despite having a diagnosis of osteoporosis by BMD (T-score ≤−2.5). One patient was hospitalized for osteomyelitis of an unrelated skeletal site and died shortly after sustaining the fracture, and the other patient was on hemodialysis and was referred but not seen in the endocrine clinic for management. Thus, overall, 23/112 (21%) patients received medication after their fracture.

A subgroup analysis of veterans aged >75 years was conducted and there were 19 men and 3 women who sustained fractures in the cohort. The majority of fractures were of the radius 6/22 (27%). Most veterans did not receive anti-osteoporosis therapy 18/22 (82%) and most did not have a BMD17/22 (77%). 

## 4. Discussion

Our study demonstrates that less than 25% of patients with a fragility fracture were prescribed a bisphosphonate in the 12 months following the fracture. BMD results seemed to drive providers’ initiation of osteoporosis treatment, as 100% of those with a T-score ≤−2.5 received or were considered for drug therapy. Although low-trauma fractures in older adults are almost always due to osteoporosis, these events commonly go unrecognized as clinical osteoporosis [19,20]. Fragility fractures confer a clinical diagnosis of osteoporosis regardless of BMD T-score [18]. Recognition and treatment of an osteoporotic fracture is paramount as the risk of a second fracture increases dramatically following the first fracture. In women, the risk of having a second hip fracture following an initial hip fracture increases six-fold, from 3.6 to 22 per 1000 person-years [21]. While medications such as calcium, vitamin D, and bisphosphonates have been shown to decrease primary and secondary fracture risk [22,23,24,25,26,27,28], these drugs remain underutilized. 

Several position statements including the Endocrine Society, American Association for Clinical Endocrinology/American College of Endocrinology, and the National Osteoporosis Foundation provide guidance for the treatment of osteoporosis that include: (1) low-trauma fracture, (2) T-score of ≤−2.5, (3) Fracture Risk Assessment Tool [FRAX] score indicating 10-year major osteoporotic fracture risk of ≥20% and/or hip fracture risk ≥3% [29,30,31]. Three years after the VA’s Office of Inspector General report, when presented with a low-trauma fracture, providers at one of the largest VA healthcare systems are still unlikely to start patients on anti-osteoporosis therapy or evaluate patients for secondary causes of osteoporosis. 

At the VHA, all patients are assigned to a Patient Aligned Care Team (PACT) team. This primary care team consists of a primary care physician, psychologist, nurse, nutritionist, and social worker. The patient benefits from this type of team approach as they receive a variety of services and different aspects of their care are distributed among the various team members. Despite this innovation, provider inertia regarding the diagnosis of osteoporosis following low-trauma fractures remains unchanged, in contrast with the outstanding performance when confronted with a BMD T-score diagnosis of osteoporosis. Our study shows that all patients who had T-scores of ≤−2.5 received or were considered for osteoporosis medication, compared to only 21% of those with an osteoporotic fracture and a T-score that did not indicate osteoporosis. Furthermore, only 2% of fracture patients were prescribed anti-osteoporosis medications without having had a prior BMD, providing insight as to why osteoporosis is often left untreated: primary care providers are more likely to act on test results than a clinical event.

Fracture Liaison Services are successful at capturing osteoporotic fractures when patients are admitted to hospitals [32]. However, not all patients require admission, especially those with fractures that do not need surgical intervention. In this study, half of the low-trauma fractures were rib, radial, and vertebral fractures, all of which rarely result in hospital admissions and thus would elude a Fracture Liaison Service. Therefore, recognition of low-trauma fractures as a clinical diagnosis of osteoporosis falls into the hands of the clinician providing primary care in the outpatient setting. The difficulties in appropriate recognition of fragility fractures as osteoporosis and delays in treatment have been documented previously, with quality improvement initiatives being established to improve treatment outcomes [33,34]. There have been interventions documenting education initiatives for providers as well as care provided by trained coordinators. In general, educational programs alone have not been successful in improving osteoporosis care [35,36,37]. Clinicians’ failure to ask about the circumstances of the fracture and to understand that any fracture resulting from a fall from a sitting or standing position is considered osteoporotic is giving rise to a large proportion of high-risk osteoporotic patients who remain untreated [12]. It is encouraging that there is acknowledgment of osteoporosis by T-score, prompting providers to initiate osteoporosis treatment.

The greatest strength of our study is that as VA is a closed healthcare system, we were able to obtain data in diagnostic and treatment rates before and after the fracture. This would not have been possible to determine in older adults receiving community care who often change healthcare plans. Our study has several limitations. First, this study was conducted at a VA facility, where there is a predominance of male patients, thus it is not representative of the demographics of the general population where osteoporotic fractures are more common in women [38]. This may pose an additional barrier to receiving treatment, since osteoporosis in men is not as widely recognized as it is in women. Education aimed at the screening and treatment of osteoporosis in men is needed to target this population. Second, the data for this study were obtained from information abstracted from medical records and chart review, which may have biased our results because of possible underreporting of information on follow-up evaluation and treatment outside of VA. Third, this is an observational study with a small sample size, which inherently limits the conclusions that may be drawn. Interestingly, the low post-fracture treatment rate of 20% demonstrated in this study is consistent with prior published data of post-fracture under-treatment [39].

## 5. Conclusions

We demonstrate that low-trauma fractures are under-recognized as osteoporotic fractures, as only 20% of patients undergo further evaluation or treatment. Conversely, when confronted with a diagnosis of osteoporosis by BMD T-score, 100% of physicians are more likely to initiate osteoporosis treatment. Low-trauma fracture assessment requires clinical judgement and assumes that providers consider that not all fractures are traumatic in nature. Under-recognition of low-trauma fractures as osteoporotic fractures leads to lack of osteoporosis treatment, which further increases fracture risk. Primary care providers’ recognition of low-trauma fractures as diagnostic of osteoporosis is key. This ensures proper assessment and management of such fractures, as they do not require hospitalization and thus fall outside the scope of a Fracture Liaison Service. Continuous education in the primary care setting is necessary to improve the recognition and medical treatment of osteoporotic fractures.

## Figures and Tables

**Table 1:** Demographics, *n* = 114 low trauma fractures.

Variable	*n* (%)
Age, mean (SD)	68 (9.5)
Gender, male (*n*, %)	105 (92)
Race/ethnicity (*n*, %)	
Non-Hispanic black	24 (21)
Non-Hispanic white	64 (56)
Hispanic white	20 (18)
Unknown	6 (5)
Comorbidities (*n*, %)	
Diabetes	21 (18)
Tobacco	17 (15)
COPD	11 (10)
Alcohol abuse	17 (11)
Seizure disorder	5 (4)
GFR < 30 mL/min per 1.73 m^2^	4 (4)

SD: standard deviation; *n*: sample size; COPD: chronic obstructive pulmonary disease; GFR: glomerular filtration rate.

**Table 2:** Fracture frequency (*n* = 114).

Fracture Type	*n* (%)
Rib	21 (18)
Radius	19 (17)
Vertebral	17 (15)
Femoral neck	17 (15)
Humerus	8 (7)
Fibula	8 (7)
Patella	7 (6)
Ulna	4 (4)
Tibia	4 (4)
Sacrum	3 (3)
Clavicle	3 (3)
Femoral shaft	3 (3)
